# Our Inconsistencies

**Published:** 1902-09-06

**Authors:** 


### Our Inconsistencies.

We often hear it said that the medical profession is sadly misunderstood by the public, and that the high standard of ethics which we set before us as our guide is by no means appreciated as it ought to be. Yet there is some excuse for the said public in its disdainful treatment of our etiquette and our rules of professional propriety, for we find but little unanimity even amongst ourselves as to the punishment to be meted out to those who defy these rules and follow their own course for their own ends. It is a perennial source of wonderment to outsiders that the medical profession should make such a point as we all do—at any rate in our public utterances—as to the impropriety of advertising. Yet with the examples which stare us in the face of the demoralising consequences of the practice, we surely are right in holding it to be an unclean thing, and in maintaining that a commandment which ought to be most implicitly obeyed by medical practitioners is :—Thou shalt not advertise ; and in saying this we are fully in accord with all responsible exponents of professional ethics from the Royal College of Physicians downwards.

The cynical outsider, however, who delights in sniggering at human weakness, is not slow to perceive and to point out how many members of our profession do their best to throw aside the fetters thus imposed upon their love of publicity, how bare-faced are the advertisements which, by one subterfuge or another, they manage to issue, or get issued for them, and what is worse still how entirely unpunished such infractions of professional rules are allowed to go.

Some remarks which we made a little while ago on this subject have led to our receiving divers complaints from medical men practising in certain watering places concerning the extent and the evil results of the open and unblushing advertisements of medical practitioners in connection with so-called "hydros."

It is all very well to say that such advertisements can do no man any good. Among his colleagues of course they can do nothing but harm, but among the great unthinking mass of the public they do have an effect, and they do lead to practice ; and what our friend the cynical outsider sniggers at most delightedly is the fact that, notwithstanding all the thunder which we hurl against them, these advertisers do not lose caste. They are members of medical societies and associations, their little puffing articles are accepted by the medical papers, and notwithstanding all the sins and wickedness of which they are guilty, the world, and even the medical world, appears to be not a penny the worse.

We have before us a weekly paper, *The English and American Gazette,* which is published in Paris and is to be found lying on the tables of many Continental hotels. It apparently appeals rather to invalid wanderers, if we may judge from the prominence given in one of its principal articles to a description of some wonderful "Professor" in London who cures all sorts of diseases by electricity.

Turning to the advertisement columns of this publication we find nearly a page devoted to a list of the members of the Continental Anglo-American Medical Society. Here they all are, these good doctors, arranged under the headings of the places in which they practise ; so that wherever an Anglo-American may happen to be, he can at once find the name of a doctor by turning to this handy list of this medical-mutual-advertising association. The worst of the matter, however, is that the men who do these things are not the sweepings of the profession, but men who hold their heads high not only among their clients but among their professional brethren. The Royal College of Physicians has absolute disciplinary powers over its Fellows and its members and yet these things are allowed to go on, not only unchecked but under the direct patronage and encouragement of its leading Fellows.

On July 31, according to the *Lancet,* the Anglo-American Continental Medical Society gave a luncheon at the Grand Hotel, Manchester, all the names mentioned as those of members present, being with one exception among those advertised in the *English and American Gazette.* Among those who, by their presence as guests, gave their sanction to the methods of this society were Sir William Broadbent, Professor Clifford Allbutt, Dr. J. F. H. Broadbent, Dr. Cholmeley, and others. Nor were these gentlemen present in an accidental manner, such as may happen to anyone during a meeting of the British Medical Association, for Sir William Broadbent responded for "The Guests," and Professor Allbutt actually proposed "The Society," to which he gave his blessing with considerable *empressement.*

What, then, are we to say when our sniggering friend points out that, notwithstanding the high aims and high standards of our profession, its members can join an advertising association like this without losing caste, that their so doing is winked at and allowed by those in high places, and that at the cost of a luncheon these erring members can obtain the sanction and even the blessing of a Regius Professor of Medicine, an ex-Censor of the Royal College of Physicians, and an editor of a leading professional organ ? What *can* we say ?

